# Phosphoprotein network analysis of white adipose tissues unveils deregulated pathways in response to high-fat diet

**DOI:** 10.1038/srep25844

**Published:** 2016-05-16

**Authors:** Alli Shaik Asfa, Beiying Qiu, Sheena Wee, Hyungwon Choi, Jayantha Gunaratne, Vinay Tergaonkar

**Affiliations:** 1 Institute of Molecular and Cell Biology, Agency for Science, Technology and Research, 61 Biopolis Drive, Singapore 138673, Singapore; 2 Saw Swee Hock School of Public Health, National University of Singapore and National University Health System, 12 Science Drive 2, Singapore 117549, Singapore; 3 Department of Anatomy, Yong Loo Lin School of Medicine, National University of Singapore, 10 Medical Dr, Singapore 117597, Singapore; 4 Department of Biochemistry, Yong Loo Lin School of Medicine, National University of Singapore, Singapore 117597, Singapore; 5 Centre for Cancer Biology, University of South Australia and SA Pathology, Adelaide, Australia

## Abstract

Despite efforts in the last decade, signaling aberrations associated with obesity remain poorly understood. To dissect molecular mechanisms that define this complex metabolic disorder, we carried out global phosphoproteomic analysis of white adipose tissue (WAT) from mice fed on low-fat diet (LFD) and high-fat diet (HFD). We quantified phosphorylation levels on 7696 peptides, and found significant differential phosphorylation levels in 282 phosphosites from 191 proteins, including various insulin-responsive proteins and metabolic enzymes involved in lipid homeostasis in response to high-fat feeding. Kinase-substrate prediction and integrated network analysis of the altered phosphoproteins revealed underlying signaling modulations during HFD-induced obesity, and suggested deregulation of lipogenic and lipolytic pathways. Mutation of the differentially-regulated novel phosphosite on cytoplasmic acetyl-coA forming enzyme ACSS2 (S263A) upon HFD-induced obesity led to accumulation of serum triglycerides and reduced insulin-responsive AKT phosphorylation as compared to wild type ACSS2, thus highlighting its role in obesity. Altogether, our study presents a comprehensive map of adipose tissue phosphoproteome in obesity and reveals many previously unknown candidate phosphorylation sites for future functional investigation.

Obesity, characterized by excess fat accumulation, is an epidemic and complex metabolic disorder caused by both life style and genetic variation^1^. Obese individuals are at a high risk for several pathological conditions including type 2 diabetes, cardiovascular diseases, and various forms of cancer^2^,^3^. Despite multiple causative and associated factors, a lifestyle change characterized by increased consumption of hypercalories is regarded as the most important contributing factor to obesity. Imbalance in energy homeostasis triggers excessive fat accumulation in the adipose tissue disrupting normal functioning of adipocytes, leading to accumulation of triglycerides within the skeletal muscles and liver as ectopic fat. Ectopic lipid, together with increased circulating free fatty acids (FFA), causes insulin resistance in various tissues, thus disrupting glucose homeostasis^4^. Obesity-associated insulin resistance is a major risk factor for diseases ranging from diabetes to cancer and involves a dynamic interplay of various cell-intrinsic, inflammatory and hormonal processes^5^,^6^,^7^. Despite this knowledge, detailed pathogenesis of the metabolic syndrome and the accompanying signaling changes remains poorly understood.

White adipose tissue (WAT) is the predominant site for storage of fat with adipocytes representing the majority cell type within this tissue. Adipocytes synthesize and store triglycerides during feeding, and upon fasting they hydrolyze and release triglycerides as FFA and glycerol^8^. Adipose tissue plays key roles in maintaining metabolic homeostasis, and hypersecretion of pro-diabetic or pro-inflammatory adipocytokines is often associated with obesity or insulin resistance^9^. Several global molecular profiling studies have been carried out previously to understand adipocyte dysfunction^10^,^11^,^12^. Recently, large-scale phosphoproteomic studies that enable simultaneous detection and quantification of thousands of phosphorylation sites on proteins has been employed to decode specific signaling events in diverse metabolic contexts^13^,^14^,^15^. In fact, one such study uncovered novel mechanisms of the AKT-mTORC2 signaling network in insulin-responsive 3T3-L1 adipocytes and pointed that insulin signaling networks were more complex than previously perceived displaying dynamic interplay among the kinases involved^16^. While kinase pathways regulate signaling output, kinase perturbations also reflect on metabolic networks since activities of enzymes are primarily regulated by their phosphorylation status at key positions. Indeed targeting upstream kinases which define major signaling nodes is one way to restore aberrations in metabolic pathways^17^,^18^. Hence we undertook this study to identify obesity-associated adipocyte phosphoproteome changes which will not only reveal molecular mechanisms of altered metabolic events, but also unravel previously unknown phosphoproteins or phosphosites that could be therapeutically targeted.

To obtain an in-depth molecular perspective of altered events in adipocytes during obesity, we performed label-free *in vivo* quantitative phosphoproteome profiling of WAT from mice fed on high-fat diet (HFD) or low-fat diet (LFD). Through comprehensive analysis of the modulated phosphoproteins, we extracted site-specific dephosphorylation events on several key enzymes involved in the lipogenic and lipolytic pathways, reflective of metabolic imbalance in lipid homeostasis during obesity. In particular, we observed phosphorylation changes on acetyl-coenzymeA synthetase (ACSS2), a key enzyme involved in lipid metabolism and energy generation. As a validation of our approach we show that the phosphorylation status on a serine residue (S263) of ACSS2 plays crucial roles in triglyceride synthesis.

## Results

### Phosphoproteome profiling of white adipose tissues from HFD- and LFD-fed mice

**Figure 1:**
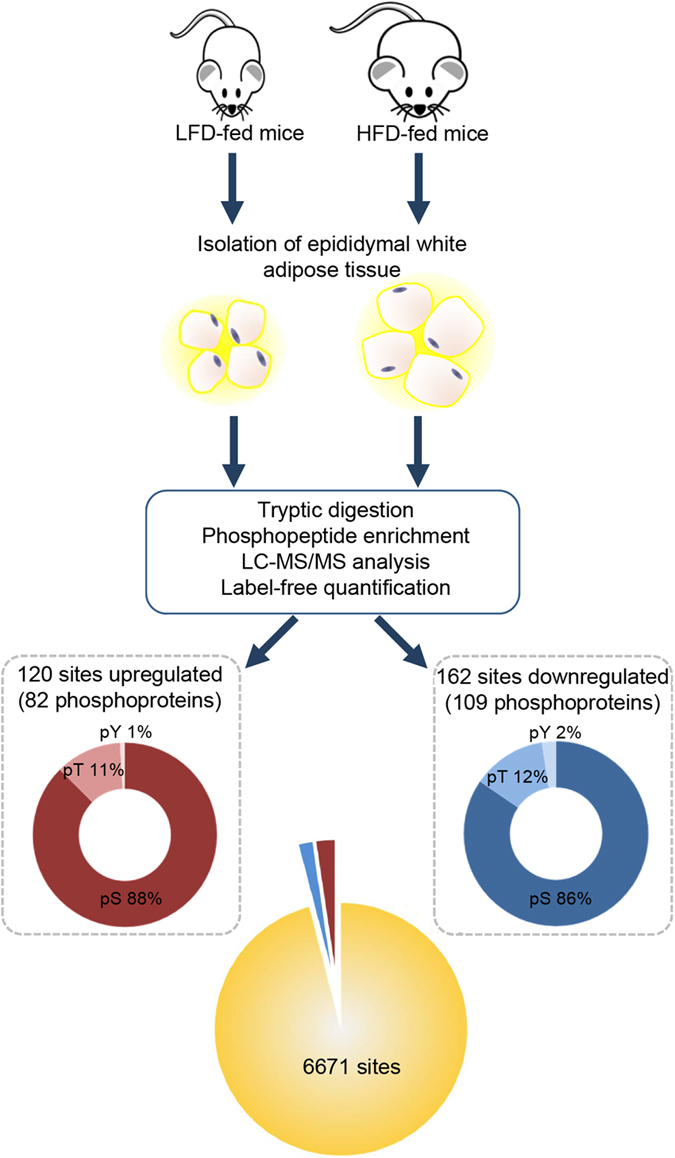
Phosphoproteome analysis of WAT upon high-fat feeding. Schema depicting the workflow used for *in vivo* label-free phosphoproteome profiling of WAT of HFD- and LFD-fed mice. Mice were maintained on HFD or LFD for 16 weeks prior to WAT isolation. Lysates obtained from three biological replicates were subjected to phosphopeptide enrichment and mass spectrometry analysis. Phosphosite localization was performed using LuciPHOR and semi-quantitation was carried out based on spectral counts. Distribution of total number of phosphosites identified at 1% FLR and differentially expressed between the HFD- and LFD-fed groups by at least two-fold at 10% FDR is summarized. The distribution of identified sites according to the S/T/Y amino acid that was phosphorylated is also shown.

We adopted a label-free quantitative mass spectrometry (MS)-based phosphoproteomic strategy to identify and characterize high-fat diet (HFD)-induced phosphorylation changes in mouse epididymal WAT (Fig. 1). To establish a HFD-induced obesity model, age- and sex- matched mice were maintained on HFD or LFD for 16 weeks. As expected, mice fed on the HFD gained more weight than those fed on the LFD, and developed obesity (Supplementary Fig. S1A). The glucose homeostasis was severely compromised in the HFD group with higher fasting glucose and serum insulin levels (Supplementary Fig. S1B,C). Consistent with obese and insulin resistance phenotypes, the HFD-fed mice displayed elevated circulating triglycerides (Supplementary Fig. S1D), impaired glucose tolerance (Supplementary Fig. S1E,F), and decreased insulin tolerance and sensitivity (Supplementary Fig. S1G,H). WAT from the two groups of mice were collected and lysates from three biological replicates (three mice in each group) were subjected to phosphoproteomic analysis as described in Methods section. Phosphopeptides were identified by database search^19^,^20^ and exact modification sites were localized using a probabilistic algorithm^21^. At 1% false localization rate (FLR), we identified a total of 7,696 phosphopeptides corresponding to 6,953 unique phosphosites on 3,115 proteins from the biological triplicates of HFD- and LFD-fed mice (Supplementary Table S1). Of these, about 89% (6,849) represented singly-phosphorylated peptides and only a small proportion, about 10% (787) and 1% (60), corresponded to doubly- and triply-phosphorylated peptides, respectively. Phosphopeptide quantification was carried out using semi-quantitation by spectral counts, and differential expression analysis revealed 120 upregulated and 162 downregulated phosphosites in response to HFD by at least two-fold (10% FDR) (Fig. 1 and Supplementary Fig. S2).

### Gene Ontology (GO) analysis of regulated phosphoproteome

**Figure 2:**
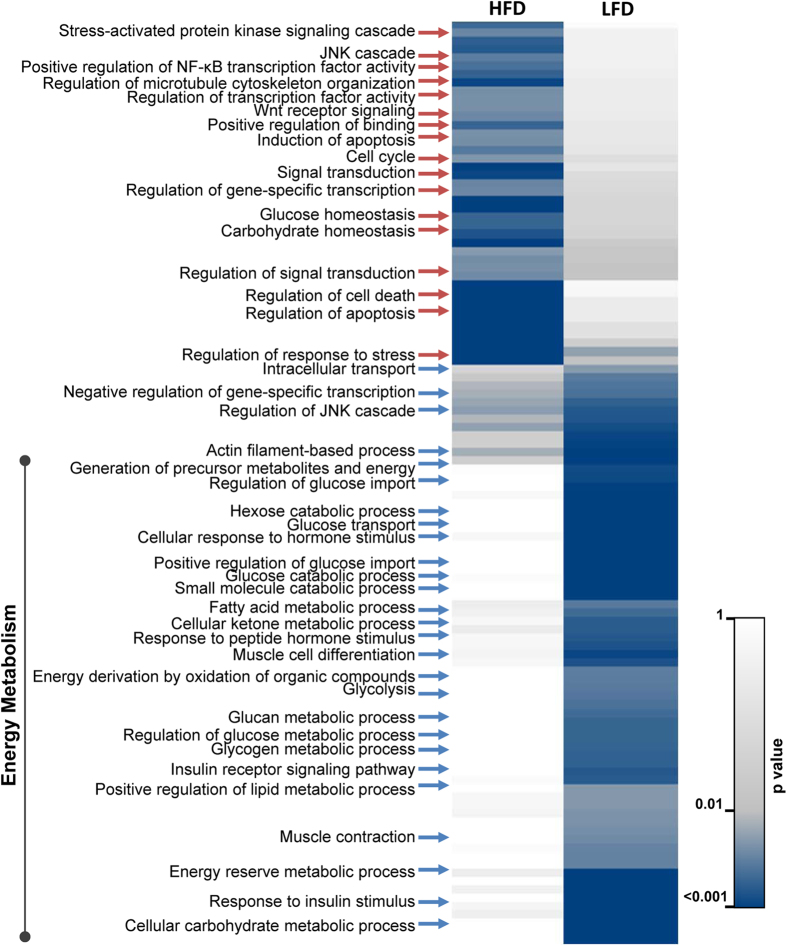
Functional analysis of phosphoproteins altered by HFD in WAT. The significantly altered phosphoproteins were enriched based on GO categorization (Biological Processes) as implemented in BINGO. Those categories with at least three proteins and significantly enriched in one of the groups with p value < 0.005 are visualized as heat map. Annotations were subjected to hierarchical clustering based on −log transformed p values to identify HFD- and LFD-specific clusters.

To gain functional insights into processes that were regulated by HFD-responsive phosphoproteins in WAT, we performed GO enrichment (Biological Process category only) for phosphoproteins with increased and decreased phosphorylation levels separately (Fig. 2). The results of this analysis highlighted distinct clusters that were exclusively enriched in response to HFD (increased phosphorylation events) or LFD (decreased phosphorylation events), indicative of unique phosphorylation signatures of WAT upon high-fat induced obesity. We found that most of the identified biological processes belonged to metabolic events including insulin receptor signaling, lipid metabolic process and glucose metabolic process known to be deregulated in diet-induced obesity. Impaired glucose tolerance and reduced insulin sensitivity are hallmarks of diabetes and obesity^22^. Accordingly, we found many proteins in glucose metabolism and insulin response with loss of phosphorylation in response to HFD, implying that these phosphorylation events may be important for metabolic regulation. Particularly, proteins involved in glucose transport, hormone response and glycogen metabolism displayed differential phosphosite regulation. We also observed selective enrichment of phosphoproteins functioning in fatty acid metabolic process in the LFD-fed mice compared to HFD-fed mice. Despite alterations of other processes such as stress signaling and apoptotic pathways with HFD, our observations underpin that phosphorylation dependent signaling is key in diet-induced metabolic pathway alterations.

### Kinases deregulated in response to HFD

Modulation of signaling response relies upon intricate wiring of protein networks and activity status of the kinases involved. As changes in phosphorylation landscape are reflective of alterations in kinase activity, we used the phosphoproteomic data to systematically identify deregulated kinases in WAT upon HFD, mainly aiming to identify kinases that regulate uncharacterized phosphorylation events in metabolic pathways.

First, we predicted kinase-substrate relationships for all the upregulated and downregulated phosphosites based on short linear motifs and protein-protein interactions as implemented in iGPS and NetworKIN computational approaches^23^,^24^,^25^. Kinase substrate enrichment analysis (KSEA) utilizing average substrate abundances was additionally performed for those kinases that were significantly enriched in both groups for confident assignment of kinases to either of the groups^26^. The predicated kinases (p value < 0.0001) are shown in Table 1. Among these kinases, AMP-activated protein kinase (AMPK), a known regulator of energy metabolism and potent drug target for metabolic diseases, was predicted to be active in LFD-fed WAT in comparison to HFD-fed WAT. AMPK signaling is known to be deregulated in obesity, and current activators are mostly specific to the AMPK complex containing regulatory β1 subunit (PRKAB1)^27^. Interestingly, we found in our dataset that phosphorylation of AMPK β2 subunit (PRKAB2) on serine 38 was upregulated in WAT of HFD-fed mice implying that β2 subunit might have unique functions in response to dietary cues. Notably, this site has been previously implicated in constituting a negative feedback loop for AMPK activity^28^. We also observed significant enrichment of targets of AKT among those phosphoproteins that displayed reduced phosphorylation in HFD-fed WAT, consistent with previous reports linking impaired AKT activation and diet-induced obesity, and also other related metabolic disorders including insulin resistance and hyperglycaemia^29^. A majority of the kinases that were predicted to be impaired in response to HFD belonged to the Ca^2+^/calmodulin-dependent protein kinase-like (CAMK-like) family of kinases, including the AMPK-related salt-inducible kinases (SIKs), sucrose non-fermenting-related kinase (SNRK), STE20-related proline alanine rich kinase (SPAK) and maternal embryonic leucine zipper kinase (MELK). Among these, SIK2 and SIK3 were significantly deregulated with reduced phosphorylation on multiple sites in our dataset. These kinases, regarded as gatekeepers of hepatic gluconeogenesis, are also known to regulate triglyceride level^30^,^31^. The other predicted dysfunctional kinase, SNRK, is known to be abundantly expressed in adipose tissue with its level negatively correlated with obesity^32^. We also observed a significant enrichment of substrates such as PAK1, PAK2 and MRCKα phosphorylated by CDC42 signaling-associated kinases in LFD-fed mice, emphasizing possible roles for such kinases in glucose and lipid homeostasis.

Substrate-based kinase prediction also revealed that the activities of certain kinases belonging to CDK subfamily (CDK10, 11 and 18), p38 MAP kinase, and casein kinase may be enhanced in response to HFD. In fact, a close homolog of predicted CDK18, PCTAIRE-1, was identified as a negative regulator of insulin-induced glucose transport in an RNA interference-based screening in adipocytes^33^. This highlights that in addition to previously reported obesity modulators such as CDK5^34^, these CDK subfamily kinases may have potentially important roles in maintaining adipocyte function and energy metabolism.

### Network of the regulated phosphoproteome during obesity

To explore the functional connectivity among the regulated phosphoproteins, we constructed an integrated protein-protein interaction network by combining interactions from STRING, Reactome Functional Interaction (Reactome FI) and GeneGo Metacore^35^,^36^. The interactions from Reactome FI and MetaCore also represent manually curated pathway-informed functional interactions. We were interested in exploring the entire spectrum of molecular interactions between our regulated phosphoproteins, and hence we combined all interactions representing both physical and functional associations (see Methods). The outcome from each database is summarized in Supplementary Table S2. The integrated network consisted of 124 nodes and 291 pairwise interactions. Since the predicted upstream kinases for each of the modulated sites upon HFD also represent meaningful functional associations, we additionally mapped these predicted kinase-substrate interactions to obtain a comprehensive functional interactome representing 151 nodes and 476 edges containing 13 predicted kinases (Supplementary Fig. S3). Analysis of the topological parameters of this comprehensive network pointed to a scale-free network based on the distribution of number of edges (degree) per node (Supplementary Fig. S4 and Supplementary Table S3). A few nodes had more edges than the average of the entire network (average = 5.8) and these ‘hubs’ likely represent crucial modulators of obesity. Around 14 nodes representing the top 10% of the degree distribution displayed high connectivity and these included kinases such as AKT2, MAPK3, PAK1, PRKCD, PKA, SIK2, transcriptional regulators such TCF7L2 and NCOR2, and enzyme involved in fatty acid biosynthesis, ACACA.

**Figure 3:**
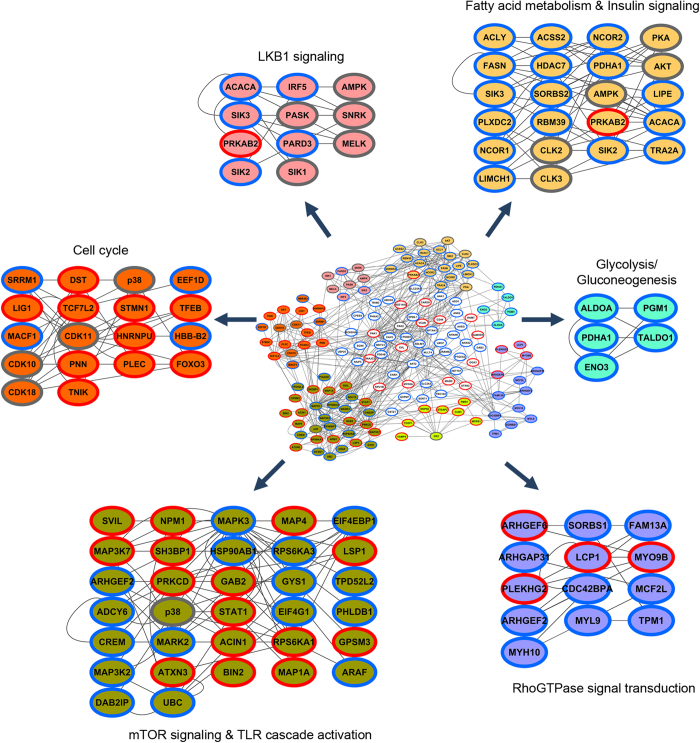
Protein-protein interaction network among altered phosphoproteins. The integrated functional network built from phosphoproteins regulated upon HFD-induced obesity in WAT. The densely connected sub-networks extracted using ClusterONE from the total network are shown in different colors. Nodes with red and blue borders correspond to upregulated and downregulated phosphoproteins, respectively. Gray border correspond to kinases mapped based on interactions from kinase-substrate prediction analysis.

Functional enrichment analysis of the network revealed components of the insulin signaling pathway suggesting that the known proteins are successfully mapped onto the network. Densely connected cluster of proteins within the network were analysed using ClusterONE, and a total of six clusters with a p value < 0.05 and one cluster with p value < 0.1 were identified^37^. Functional roles of each of these modules were assessed by ontology/pathway analysis, and the extracted clusters are shown in different colors in Fig. 3. The cluster shown in yellow comprised of functional associations between proteins primarily involved in energy homeostasis. This cluster included components of lipid metabolism such as ACACA, FASN and LIPE, and these enzymes were found to be in close association with kinases such as AKT, AMPK, PKA and SIK2 that are known to regulate such processes. Apart from these, transcriptional modulators such as nuclear receptor corepressors (NCOR1 and NCOR2) and histone deacetylase (HDAC7) also formed close-knit associations with this network underscoring their possible roles in regulating energy metabolism. The cluster shown in blue represented proteins involved in glucose metabolism. It is interesting to note that most proteins in both these clusters exhibited dephosphorylation during HFD-induced obesity. The cluster shown in pink (p < 0.1) contained components of LKB1 signaling that also regulate ACACA. Components of mTOR signaling pathway such as RPS6KA3, RPS6KA1, EIF4EBP1 were found in the cluster shown in green in close proximity to proteins involved in Toll-like-receptor (TLR) signaling. Other clusters identified represented those functioning in RhoGTPase signal transduction for cytoskeletal remodelling, and those regulating the cell cycle^38^. These clusters contained phosphoproteins that displayed both increased and decreased phosphosite abundance. Our network analysis thus highlighted the involvement of other components or modules that may be important in co-operating to metabolically rewire the cellular physiology during diet-induced obesity. Indeed co-operativity between very distinct signaling cascades can drive important cell fates^39^.

**Figure 4:**
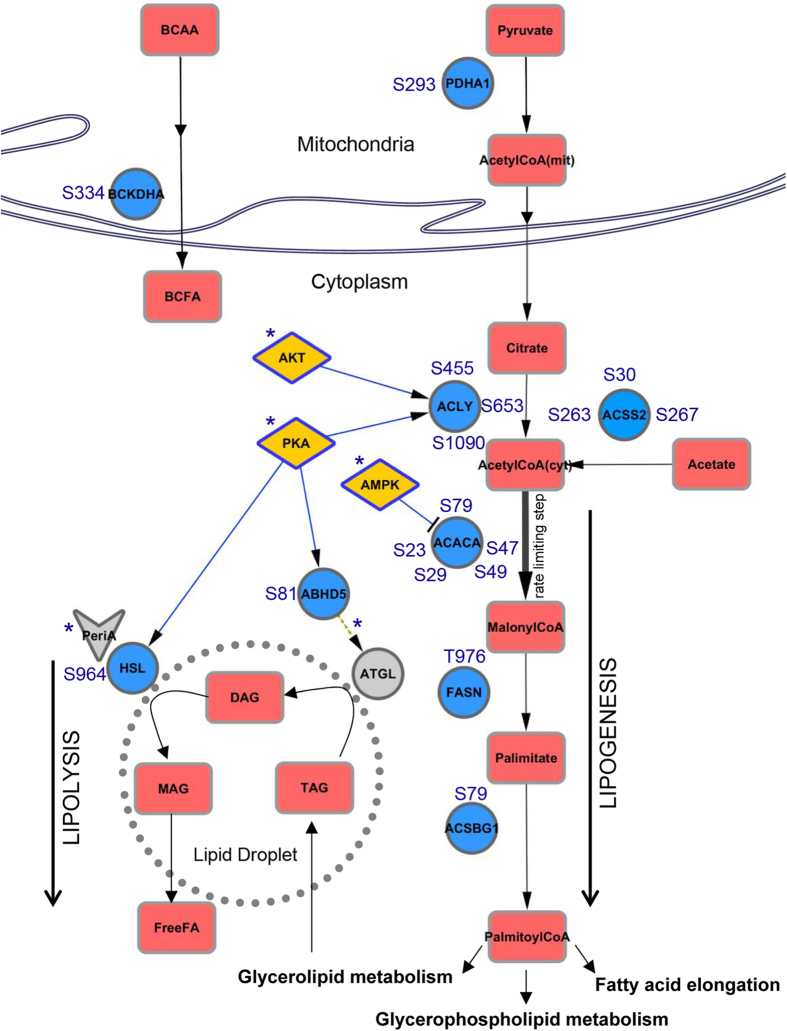
Altered phosphorylation of lipid metabolic pathway enzymes upon HFD. Enzymes with decreased phosphorylation upon HFD were mapped onto known lipid metabolic networks as annotated in the KEGG database. Metabolic links with kinases were based on curated literature-based evidences. Enzymes, metabolites and kinases are represented by circles, rectangles and diamonds, respectively. HFD-altered enzymes are shown in blue, kinases are shown in yellow and other associated proteins are shown in gray. Literature-derived regulatory roles have been indicated with asterisk (‘*’).

The most striking feature in the WAT phosphoproteome signature, also evident from our network (cluster yellow and blue), was the selective enrichment of metabolic enzymes in the downregulated phosphoproteins. Thus, we focused on such dephosphorylation events associated with metabolic pathways, particularly lipid metabolism. The fold changes associated with the differentially regulated unique phosphopeptides from these metabolic enzymes are shown in Table 2. Pathway analysis suggested that these enzymes function in glucose metabolism pathways including glycolysis, gluconeogenesis and glycogen storage, as well as lipid metabolism pathways including fatty acid and triglyceride biosynthesis (Supplementary Fig. S5). To unravel the metabolic context, we mapped the deregulated components onto known metabolic networks from Kyoto Encyclopedia of Genes and Genomes (KEGG) PATHWAY database, and curated additional evidences regarding the enzyme activity status and upstream kinases from literature. The schema for lipogenic and lipolytic pathway is shown in Fig. 4. Among them, we noticed acetyl-coA carboxylase-1 (ACACA), the rate-limiting enzyme in fatty acid biosynthesis that mediates the formation of malonyl-coA from acetyl-coA with five downregulated phosphorylation sites. The key phosphorylation site at serine 79 that inhibits the enzyme activity^40^,^41^ was significantly downregulated (~9-fold), suggestive of an activation of ACACA in high-fat fed WAT. This site is also a direct target for AMPK and hence fits well with our kinase-substrate prediction results that suggested reduced activity of AMPK in response to high-fat feeding. It is also interesting to note that a decrease in phosphorylation of serine 29 has been previously observed with knockdown of AMPK-related SNRK^32^, another predicted kinase from our analysis, implying that this site may have additional regulatory roles in maintaining lipid homeostasis.

Upstream of ACACA, we observed downregulated phosphosite at serine 293 of pyruvate dehydrogenase E1 component subunit alpha (PDHA1) with HFD. Dephosphorylation of this site increases the enzyme activity^42^, thus enhancing mitochondrial acetyl-coA production that can be routed to TCA cycle for oxidation (in mitochondria) or stored as fats (in cytoplasm). We also observed multiple sites with reduced phosphorylation on ATP-citrate lyase (ACLY) and cytoplasmic acetyl-coenzyme A synthetase (ACSS2) that mediate production of cytoplasmic acetyl-coA important for priming fatty acid synthesis. The multiple alterations on cytoplasmic acetyl-coA-forming enzymes complemented with increased activity of rate-limiting ACACA, suggest possible dysregulation of fatty acid biosynthesis pathway in adipocytes during obesity.

Furthermore, downstream of ACACA, the phosphosite abundance on fatty acid synthase (FASN) that acts as the second major enzyme of fatty acid biosynthesis was significantly altered. This specific site, threonine 976, has also been previously reported in brown adipose tissues^43^, stressing that it may have important implications in adipose-specific functions. Additionally, an acyl-coA synthetase enzyme belonging to the bubblegum family (ACSBG1) that mediates activation of long-chain or very-long-chain fatty acids also displayed reduced phosphorylation at a novel site. Though little is known about this particular isoform that is preferentially expressed in brain and testis^44^, the enzyme has potential implications in enhancing the uptake of fatty acids, trapping them and routing them for downstream lipid metabolism including triglyceride synthesis like their counterparts^45^. Apart from these lipogenic enzymes, we also observed loss of phosphorylation on branched chain keto acid dehydrogenase E1 alpha (BCKDHA) that is involved in the catabolism of branched-chain amino acid (BCAA) and synthesis of branched-chain fatty acid (BCFA), indicative of a deregulation of BCAA metabolism during high-fat feeding.

Key proteins involved in the lipolytic pathway were also observed with decreased phosphorylation. Indeed, we identified dephosphorylation of the rate-limiting hormone-sensitive lipase (HSL) at the major activity regulatory site serine 964 (serine 660 in rat) that is known to be phosphorylated by PKA^46^, implying a decrease in lipolytic activity of HSL in WAT during high-fat feeding. Also, a substantial reduction in the phosphosite abundance of CGI-58 (also known as ABHD5), a coactivator of adipose triglyceride lipase (ATGL)^47^, was observed. PKA mediated phosphorylation on CGI-58 has been previously shown to increase CGI-58 availability for ATGL co-activation^48^, and the novel ABHD5 site we quantified is also predicted to be phosphorylated by PKA, suggesting that ATGL activity may be compromised in adipocytes during high-fat feeding. This is consistent with previous reports that suggest reduced ATGL and lipase activity in adipose tissues during obesity, and underscores the importance of phosphorylation in coordinating lipolytic response^49^.

Taken together, our analysis shows a shift in the balance between lipogenesis and lipolysis pathways in WAT as a result of changes in the kinase signaling mechanisms during high-fat feeding.

### Functional implications of novel ACSS2 phosphorylation site

**Figure 5:**
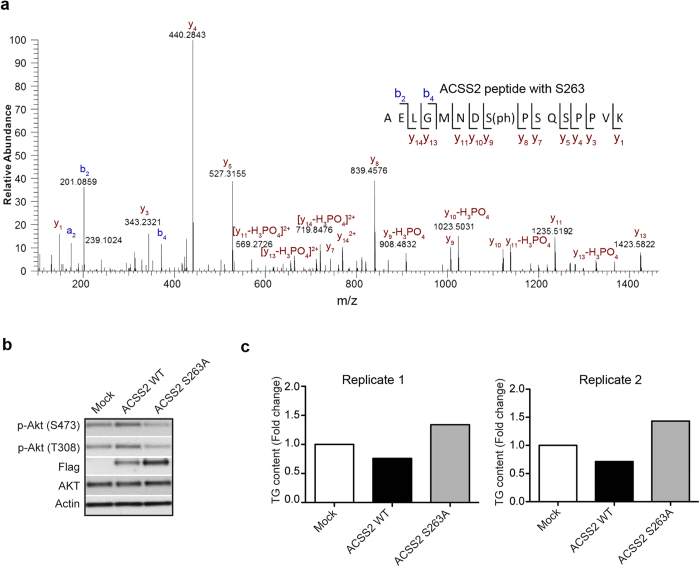
HFD altered ACSS2 phosphorylation affects insulin signaling and triglyceride level. **(a)** MS/MS spectra validating the phosphorylation of ACSS2 at serine 263 **(b)** WT ACSS2 and mutant ACSS2 S263A were expressed in 3T3-L1 adipocytes and treated with 1 μM Dex followed by insulin stimulation. Western blot analysis of insulin resistance measured using pAKT (S473) and pAKT (S308). Flag indicates expression levels of WT ACSS2 and ACSS2 S263A mutant. Actin served as loading control (n = 2 biological replicates). (**c**) Fold increase in triglyceride level in 3T3-L1 adipocytes overexpressing WT ACSS2 and mutant ACSS2 S263A upon Dex treatment (n = 2 biological replicates).

To investigate whether the phosphosites we observed in our phosphoproteome analysis have functional roles in regulating insulin signaling or lipid metabolism, we set up a cell model that mimics insulin resistant status. Accordingly, 3T3-L1 cells were cultured in conditioned media for 8 days, differentiated into adipocytes, and treated with l0 nM insulin (CI) or 1 μM Dexamethasome (Dex) for 16 hours. Insulin signaling was monitored by challenging the adipocytes with acute insulin or metformin. While CI and Dex both led to impairment of insulin signaling as observed by decreased AKT phosphorylation status (Supplementary Fig. S6A), Dex could additionally impair AMPK signaling as revealed by reduced phosphorylation of AMPK and its downstream substrate ACC (acetyl-coA carboxylase) upon metformin treatment (Supplementary Fig. S6B). Dex treated adipocytes further displayed elevated triglyceride content, suggesting that the cell model can represent a suitable *in vitro* insulin resistance model with high lipid content (Supplementary Fig. S6C). Since many of the metabolic enzymes in the lipid synthesis pathway were dephosphorylated, we speculated if these sites could have unrealised functional implications in glucose or lipid homeostasis. We focused on enzyme ACSS2 that catalyzes the synthesis of cytoplasmic-acetyl-coA essential for lipid synthesis and also displayed reduced phosphorylation on multiple sites upon high-fat feeding. To study the functional consequence of site S263 that was reduced by about 5-fold, we overexpressed ACSS2 WT and ACSS2 S263A (mutant with serine 263 to alanine substitution) in 3T3-L1 adipocytes separately, and checked for compromise of insulin signaling as determined by phosphorylation of AKT (S308 and S473) in a Dex treated model following insulin stimulation (Fig. 5a). We observed that overexpression of mutant ACSS2 S263A led to decreased phosphorylation of AKT on both sites compared to that of ACSS2 WT and mock control, showing that ACSS2 S263phosphorylation plays an important role in regulating insulin signaling (Fig. 5b). We further checked the triglyceride level in 3T3-L1 adipocytes that overexpress ACSS2 WT or mutant ACSS2 S263A (Fig. 5c). ACSS2 S263A overexpression led to a pronounced increase in triglyceride level by about 2-fold as compared to ACSS2 WT. Altogether, these experimental observations suggest that S263 phosphorylation of ACSS2 plays a crucial role in maintaining triglyceride level and in modulating insulin-induced AKT phosphorylation during obesity, thus highlighting it as a potential target in regulating glucose and lipid homeostasis.

## Discussion

In this study, we provide a system-wide quantitative atlas of phosphorylation changes in WAT in response to HFD-induced obesity using label-free phosphoproteomics. In addition to reflecting known signaling events implicated in obesity, such as aberrant insulin signaling and associated insulin resistance, the snapshot of the adipocyte phosphoproteome points to intricate rewiring of metabolic network in a phosphorylation-dependent manner. Our dataset uncovered an array of phosphorylation changes on several key enzymes involved in lipid and glucose homeostasis, implying that these events may underlie important functional consequences in adipocyte reprogramming during obesity or insulin resistance. Though other large-scale proteomic studies have been previously performed in adipose tissues^10^,^16^,^50^, our phosphoproteome profiling recapitulates actual changes that occur *in vivo* during obesity. Our atlas revealed several uncharacterized candidate sites in the context of obesity and hence is a valuable resource for elucidating molecular events accompanying HFD-induced adipocyte dysfunction, obesity and insulin resistance.

Metabolic disorders are characterized by dysregulated insulin response, impaired glucose homeostasis and insulin resistance. Insulin resistance is manifested by aberrant trafficking of insulin-responsive glucose transporter GLUT4 (known as SLC2A4) to the plasma membrane, and this translocation is dependent on the phosphorylation of TBC1D4^51^,^52^. We identified both these proteins with loss of phosphorylation suggestive of defects in insulin-stimulated signaling and subsequent glucose import during HFD-induced obesity. In addition, we also observed reduced phosphorylation on two sites in AKT1S1, a known downstream substrate of AKT. AKT1S1 (known as PRAS40) is a known modulator of mTOR signaling. A reduction in phosphorylation at another site (threonine 246 in human) has been previously observed upon HFD in several tissues^53^. AKT is the key driver of insulin signaling and we observed reduced phosphosite abundance on threonine 450 which has been proposed to prime the full activation of AKT^54^. Along with the insulin signaling components, those belonging to mTOR pathway also showed altered phosphorylation profile with HFD. Together, these suggest that high-fat feeding perturbs normal AKT-mTOR signaling machinery which could be therapeutically targeted^55^.

Despite the wealth of data that can be acquired from a typical phosphoproteome experiment, the elucidation of the overall functional consequences of phosphorylation events is quite challenging. The phosphoproteome landscape as such is reflective of modulated kinase signaling events and linking the altered kinase signaling events in the context of protein or metabolic networks can therefore reveal activated or suppressed molecular events during obesity. Using our dataset, we predicted several upstream kinases that are likely affected during high-fat feeding. Apart from the well-documented AKT and AMPK-family of kinases, we predicted several others including MRCKα or PAK 1/2 which are not clearly understood in the context of obesity. These kinases function as a part of the CDC42 signaling, and a role for CDC42 in the insulin signaling cascade and glucose translocation has been previously suggested^56^. We observed other components of the RhoGTPase signaling forming densely connected functional modules in our network. Also, we predicted PKA to be downregulated in WAT during obesity. PKA signaling plays a vital role in regulating energy homeostasis, and a tissue-specific differential regulation of PKA activity has been suggested with induction of PKA ameliorating obesity in certain cell types^57^. Our prediction resonates well with recent studies that have observed rescue of diet-induced obesity and insulin resistance upon disruption of RIIβ PKA regulatory subunit (expressed primarily in adipose tissues and brain), which consequentially increases basal PKA activity^58^. We also observed increase in phosphorylation of kinases on sites of unknown functional consequences upon HFD. One among them is TAK1 that plays regulatory roles in inflammation and metabolism via tumor necrosis factor (TNF), toll-like receptor, and interleukin 1 signaling pathways. Though a functional relationship between TAK1 and AMPK has been previously established, their potential crosstalk in the context of metabolic disorders like obesity remains to be elucidated^59^.

To respond to various nutritional statuses, cycles of lipogenesis and lipolysis are regulated to maintain energy homeostasis. The balance between these processes controls flux of fatty acid and ectopic lipid accumulation; however, there have been contradictory observations with respect to their status in the adipose tissue^60^,^61^,^62^. For ectopic fats, while some studies claim that increased triglycerides in muscle may lead to insulin resistance, others suggest increased triglycerides during exercises correlate with insulin sensitivity^63^. In our dataset, we observed hypophosphorylation on several enzymes implicated in the lipogenesis pathway, including ACACA (on the key activity site), and FASN with high-fat feeding. Accumulation of cytoplasmic acetyl-coA primes the synthesis of fatty acids, and interestingly, we observed reduced phosphorylation on both enzymes, ACLY and ACSS2, that catalyses its formation. We posit that the concerted effect of such dephosphorylation on metabolic enzymes may lead to metabolic rewiring and increase triglyceride level during obesity. Indeed, we found one such novel site on ACSS2 at serine 263 that is important for maintaining triglyceride level stressing on its possible roles in regulating lipogenesis/lipolysis balance. From our network, we identified many components of the lipolysis and lipogenesis pathway forming close connections with each other, and also with key metabolic kinases such as AKT, AMPK and PKA (yellow cluster in Fig. 3), suggesting potential regulatory associations between different pathways. Confirming to that, we found that phosphorylation of serine 263 in ACSS2 (also in the same cluster) regulates insulin-stimulated AKT phosphorylation, underscoring potential cross-talk between enzyme phosphorylation and cell-signaling rewiring. Thus, along with the altered kinase signaling activities, the observed loss of phosphorylation on several of the lipogenic enzymes including the validated ACSS2 lead us to speculate an increase in adipocyte lipogenic output with high-fat feeding. The offset in the lipogenic-lipolytic balance may be a consequence of aberrant kinase signaling that propagates via changes in enzyme phosphorylation.

In summary, this study expands our understanding of the molecular mechanisms modulated during obesity by providing an in-depth systems level analysis of the altered phosphorylation profile. Our global phosphoproteome profile stresses on a phosphorylation-dependent modulation of metabolic output, and also unveils several uncharacterized phosphosites that may play important regulatory roles in glucose and lipid metabolic pathways. We believe that an extensive phosphoproteomic interrogation of various tissues in different metabolic milieu will provide a holistic view of the molecular aspects that underlie complex metabolic disorders like obesity, and reveal context-dependent or tissue-specific regulation of proteins maintaining energy homeostasis.

## Methods

### Reagents

Insulin levels were measured using Rat/Mouse Insulin ELISA kit (Merck). Triglycerides and FFAs were measured by Triglyceride Quantification Colorimetric/Fluorometric Kit (Biovision). pLenti-GFP-Zeo was purchased from Addgene. Mutants were generated by using QuikChange Lightning Site-Directed Mutagenesis Kit (Agilent). AKT, pAKT, pAMPK, and pACC antibodies were obtained from Cell Signaling Technology.

### Animal care and diet regimen

Mice were kept on a 12-hour light-dark cycle and fed on either a standard rodent chow (NCD, 18% kcal from fat, Harlan), or a high-fat diet (HFD, 60% kcal from fat, Research Diet D12492). HFD was initiated at 2 months of age for 4 months. Body weights were monitored every other week. All procedures involving animal experimentation were approved by the Institutional Animal Care and Use Committee (IACUC) of A*STAR. Principles of laboratory animal care (NIH publication no. 85–23, revised 1985; http://grants1.nih.gov/grants/olaw/references/phspol.htm, accessed 1 January 2012) were followed.

### Ethical approval

Animal experiments were conducted according to guidelines of the Agri-Food and Veterinary Authority of Singapore.

### Glucose and insulin tolerance tests

These physiology tests were performed essentially as previously described^64^. Briefly, for glucose tolerance tests (GTT), age-matched mice were starved for 18 hours, and blood samples were obtained at 0, 15, 30, 60, and 120 minutes after intraperitoneal injection of D-glucose at 2 g/kg body weight. For insulin tolerance tests (ITT), mice were starved for 4 hours and injected intraperitoneally with recombinant human insulin at 1 U/kg body weight (Actrapid HM Penfill, NovoNordisk, Copenhagen, Denmark), with blood sampling at 0, 15, 30, 60 and 120 minutes after injection. Blood glucose values were determined by measuring tail-vein blood samples using Accu-Chek Advantage blood glucose meter (Roche Diagnostics GmbH, Mannheim, Germany).

### Cell culture

3T3L1 pre-adipocyte cell line was obtained from the American Type Culture Collection. Cells were cultured in DMEM supplemented with 10% calf serum. 3T3L1 pre-adipocytes were differentiated into 3T3L1 adipocytes as previously described^65^. For induction of insulin resistant model *in vitro*, differentiated 3T3L1 adipocytes were treated with 10 nM insulin or 1 μM Dexamethasone (Dex) for 16 hr.

### Statistical analysis

Statistical analyses were performed by Graphpad Prism 5.0. Differences between the two groups of mice that were fed on HFD or LFD were calculated using an unpaired Student’s t-test (two-tailed). P value <  0.05 was considered statistically significant.

### Reduction, alkylation and digestion

Epididymal white adipose tissues (WAT) of the HFD-fed or LFD-fed mice (1.3 mg) was lysed in 9 M urea buffer consists of 20 mM HEPES (pH 8), 9 M urea, 1 mM activated sodium orthovanadate, 2.5 mM sodium pyrophosphate, and 1 mM ß-glycerophosphate, yielding 1.3 mg protein. Reduction of the protein was carried out by the addition of 1/10 volume of 55 mM DTT to the cleared cell supernatant and incubation for 30 minutes at room temperature. This was followed by protein alkylation performed by the addition of the same volume of 120 mM iodoacetamide and incubation for 30 min at room temperature in the dark. The samples were then diluted using 20 mM Hepes (pH8) to a final concentration of 6 M urea before the addition of LysC (Wako 129-02451) with a LysC to protein ratio of 1:100 and incubation at 37 °C overnight. The samples were further diluted to a final concentration of ~1 M urea using 20 mM Hepes (pH8). Sequencing-grade trypsin (Promega) was added with a trypsin to protein ratio of 1:50 (w/w) and digestion was carried out for 4 hours at 37 °C, The resultant peptide solution was cleaned up using an Empore^TM^ C18-SD Cartridge (3 M Empore) and the amount of peptide obtained was measured using Nanodrop (ThermoScientific).

### IMAC beads preparation

IMAC beads were prepared as described by Ficarro *et al*. with slight modifications^66^. Briefly, 200 uL Ni-NTA agarose conjugates (Qiagen) were rinsed with 800 μl MilliQ water 3 times before stripping nickel off the beads by incubation with 800 μl of 100 mM EDTA (pH 8.0) for 30 minutes at room temperature with end-over-end rotation. Residual nickel was removed by rinsing with 800 μl MilliQ water 3 times. This was followed by incubation with 800 μl of a 100 mM iron chloride solution for 30–45 minutes. The resin was washed with 800 μl MilliQ water 3 times, followed by rinsing with 800 μl 80% acetonitrile/0.1% trifluoroacetic acid 4 times.

### Phosphopeptide enrichment

650 μg tryptic peptides were reconstituted in 650 μL 50% acetonitrile/0.1% trifluoroacetic acid, before being diluted 1:1with 100% acetonitrile/0.1% trifluoroacetic acid. Phosphopeptide enrichment was carried out by incubating the tryptic peptides with 10 μL IMAC beads for 30 minutes with end-over-end rotation. The beads were then loaded onto a self-packed C18 StageTip^67^, which was pre-conditioned with 200 uL methanol, pre-washed with 100 uL 50% acetonitrile/0.1% formic acid and pre-equilibrated with 200 uL 1% formic acid. After the IMAC beads were loaded onto the stage tip, washing of the beads was performed using 100 μL 80% acetonitrile/0.1% trifluoroacetic acid and 200 uL 1% formic acid. Elution of phosphopeptides from the IMAC beads onto C18 membranes on the stage tip was carried out using 4 × 70 uL 500 mM dibasic sodium phosphate (pH 7.0). The C18 membranes were then washed using 200 uL 1% formic acid. The phosphopeptides were stored on the stage tip until they were ready to be analysed on LC-MS. Elution of phosphopeptides from the C18 membranes was carried out with 60 μL 50% acetonitrile/0.1% formic acid, followed by drying using a speedvac and reconstitution in 24 μL of 0.1% formic acid.

### LC/MS analysis

Reconstituted peptides were analysed on an EASY-nLC 1000 (Proxeon, Fisher Scientific) coupled to a Q-Exactive (Thermo Fisher Scientific). Peptides were first trapped onto a C18 pre-column and then separated on a 50 cm analytical column (EASY-Spray Columns, Thermo Fisher Scientific) at 50 °C. A 245 min gradient ranging from 0 to 40% acetonitrile/0.1% formic acid was used. This was followed by a 10 min gradient ranging from 40 to 80% acetonitrile/0.1% formic acid and remained at 80% acetonitrile/0.1% formic acid for 10 min. Survey full scan MS spectra (m/z 310–2000) were acquired with a resolution of 70k, an AGC target of 3 × 10^6^, and a maximum injection time of 10 ms. The twenty most intense peptide ions in each survey MS scan with an intensity threshold of 10 k, underfill ratio of 1% and a charge state ≥2 were isolated in succession with an isolation window of 2 Th to a target value of 50 k, maximum injection time of 50 ms and fragmented by high energy collision dissociation using a normalized collision energy of 25% in the high energy collision cell. The MS/MS was acquired with a resolution of 17.5 k and a starting m/z of 100. A dynamic exclusion with exclusion duration of 15 s was applied.

### Database search and high-confidence peptide identification

Database search was performed with X!Tandem^19^ against the Uniprot mouse database appended with reverse sequences (as of May 10th). The search parameters were: precursor mass tolerance (MS1) between −10 ppm and 20 ppm, and fragment tolerance (MS2) 0.02 Da; carbomidomethylation (C: 57.02146) as fixed modification, and oxidation (M: 15.99491) and phosphorylation (S/T/Y: 79.9663) as variable modifications; minimum parent m/z was 600–4000; spectra with 6 to 50 peaks with no noise suppression. The search results were further processed to select high-confidence phosphopeptide identifications using the Trans-Proteomic Pipeline (TPP)^20^,^68^ with the default option -OpdAEwt –drev –PPM.

### Phosphorylation site localization

To perform semi-quantitation by spectral counting at individual phosphorylation sites, site localization algorithm LuciPHOr^21^ was applied to the results with the following options: high mass accuracy option for model fitting; TPP PeptideProphet probability 0.95 for feature learning in the positive set; MS2 tolerance 0.02 Da; neutral loss (forward and decoy) of H_3_PO_4_ (97.97690); decoy site localization mass 79.966331. Scoring was performed for all peptides with initial PeptideProphet probability 0.5 and above in charge state 2+ and 3+.

### Spectral counting and differential phosphopeptide expression analysis by QSPEC

For the peptides with false site localization error rate (FLR) below 1%, the number of MS/MS spectra supporting phosphorylation on each candidate residue (S/T/Y) was counted to represent the “spectral counts” of each site. Spectral count-based tool QSPEC was used to perform differential expression analysis for each phosphosite, controlling the FDR at 10%^69^.

### GO analysis

For analysing the functional enrichment across the phosphoproteins, the significantly altered phosphopeptides with upregulated or downregulated sites upon high-fat feeding were categorized based on GO Biological Process using BINGO functional annotation tool as implemented in Cytoscape. The GO terms for each of the HFD- and LFD- fed groups were filtered to retain only those categories that were significantly enriched with p value < 0.005 and contained at least three representing genes in either of the analysed groups. The filtered p values were subjected to hierarchical clustering based on Euclidean distance and average linkage, and visualized as a heat map. Pathway annotation for metabolic enzymes was retrieved from Reactome pathway database.

### Kinase-substrate prediction

To predict potential kinase-substrate relationships and thus the kinase activity in the WAT of HFD- and LFD-fed mice groups, both iGPS (version 1.0) and NetworKIN (version 3.0) algorithm were used^23^,^24^,^25^. iGPS classifies protein kinases into hierarchies and makes predictions on the hypothesis that similar short linear motifs are phosphorylated by similar protein kinases^25^. Additionally, contextual information based on known protein-protein interactions is used to maximise true-positive hits. The significantly altered upregulated and downregulated peptides were input as PhosPEP format and predictions were made with a false positive rate (FPR) of 10% for serine-threonine kinases. Since, multiple kinases can potentially phosphorylate the same sites, the kinase-substrate relationships were filtered to include only those kinases that were detected in our WAT phosphoproteome dataset. We thus focused only on those 167 kinases with their isoforms for subsequent analysis. Statistical enrichment was performed using hypergeometric test and kinase-substrate relationships of all the detected phosphopeptides were used as background for iGPS. The NetworKIN algorithm also works in a similar fashion wherein the probabilities derived from NetPhorest atlas for kinase consensus motifs and proximity-scores for protein-protein interactions are combined to obtain NetworKIN scores represented as likelihood ratios^24^. The mouse phosphopeptides were matched to their human counterparts and predictions for the differentially expressed phosphosites were obtained in the human background. To gain confidence in the predicted outcomes, the kinase-substrate relationships of the altered phosphopeptides were filtered to include only those with a NetworKIN score >2 or NetPhorest probability >0.1 prior to statistical enrichment by hypergeometric distribution. Only those kinases that were enriched with a p value cut-off less than 10^−4^ were considered significantly altered. For those kinases that were predicted to be significantly enriched both in the upregulated and downregulated substrate groups, kinase-substrate enrichment analysis based on average abundances (log_2_ fold change) of phosphorylated substrates was additionally performed to assign the activity of the kinase to either the HFD- or LFD-fed groups.

### Network Analysis

Protein-protein interactions as implemented in STRING^36^, Reactome Functional Interaction (Reactome FI), and GeneGO MetaCore were used to explore the functional connectivity between the altered phosphoproteins. The upregulated and downregulated phosphoproteins that were differentially regulated were loaded into STRING and all pairwise interactions were downloaded. STRING reports medium confidence interaction pairs with a combined score >0.4. For our analysis we increased this threshold to 0.6 to retain higher confidence interactors. Apart from interactions retrieved from STRING, we additionally extracted pathway-based functional interaction from Reactome FI as implemented in Cytoscape visualization tool and direct interactions from MetaCore software suite^35^. The interactions reported in both these tools are manually curated based on experimental evidence, and hence all the interactions obtained were retained for analysis. Only interactions between the regulated phosphoproteins were retained and no linker candidates were included. All the interactions obtained from the above resources were parsed into Cytoscape as a SIF (simple interaction file) and merged with each other to represent an integrated functional network. Redundant entries were removed by excluding all duplicate interactions. Furthermore, significantly modulated kinases as predicted by iGPS or NetworKIN (Table 1) were mapped onto the merged network to obtain a comprehensive interactome that explores all possible functional associations between the regulated phosphoproteins. The network properties were explored using ‘Network Analyzer’ in Cytoscape. Densely connected phosphoprotein clusters were identified using ClusterONE in Cytoscape^37^. Functional enrichment in each of the clusters was performed using DAVID or Reactome pathways.

For metabolic network mapping, the substrates, products and pathways associated with each enzyme was manually obtained from KEGG and the schema was constructed using components of the pathway map corresponding to ‘Fatty acid biosynthesis’, ‘Glycerolipid metabolism’ and ‘Citrate cycle’. Known regulations such as upstream kinases and functional consequences were obtained through literature. Finally, all the information was visualized in a schematic manner.

### Data availability

The mass spectrometry proteomics data have been deposited to the ProteomeXchange Consortium (http://proteomecentral.proteomexchange.org/) via the PRIDE partner repository with the dataset identifier PXD002927.

## Additional Information

**How to cite this article**: Alli Shaik, A. *et al*. Phosphoprotein network analysis of white adipose tissues unveils deregulated pathways in response to high-fat diet. *Sci. Rep.* **6**, 25844; doi: 10.1038/srep25844 (2016).

## Supplementary Material

Supplementary Information

Supplementary Table S1

Supplementary Table S2

Supplementary Table S3

## Figures and Tables

**Table 1:** Kinases predicted to be regulated in WAT in response to high-fat feeding.

Kinase	Kinase Group	p Value	Active in	Source
CDK11	CMGC/CDK	1.31E-07	HFD	iGPS
CDK10	CMGC/CDK	1.38E-07	HFD	iGPS
CDK18	CMGC/CDK	1.92E-06	HFD	iGPS
CK2alpha	CK2_group	7.27E-08	HFD	NetworKIN
p38 (MAPK11)	p38_group	1.71E-07	HFD	NetworKIN
p38 (MAPK12)	p38_group	9.63E-05	HFD	NetworKIN
p38 (MAPK13)	p38_group	1.20E-05	HFD	NetworKIN
AKT2	AGC/AKT	2.62E-06	LFD	iGPS
PKACb	AGC/PKA	8.50E-06	LFD	iGPS
SIK	CAMK/CAMKL	4.58E-08	LFD	iGPS
MELK	CAMK/CAMKL	6.08E-07	LFD	iGPS
SIK3	CAMK/CAMKL	6.08E-07	LFD	iGPS
STK39	CAMK/CAMKL	2.07E-06	LFD	iGPS
SIK2	CAMK/CAMKL	1.98E-07	LFD	iGPS
SNRK	CAMK/CAMKL	2.72E-07	LFD	iGPS
AMPKa1	CAMK/CAMKL/AMPK	9.32E-05	LFD	iGPS
AKT1	AGC/AKT	6.52E-08	LFD	NetworKIN
PKAalpha	AGC/PKA	1.58E-11	LFD	NetworKIN
PKAbeta	AGC/PKA	3.58E-10	LFD	NetworKIN
PKAgamma	AGC/PKA	1.80E-10	LFD	NetworKIN
CLK2	CLK group	2.42E-06	LFD	NetworKIN
CLK3	CLK group	1.59E-05	LFD	NetworKIN
MRCKa	DMPK group	1.21E-12	LFD	NetworKIN
PAK1	PAK group	5.38E-12	LFD	NetworKIN
PAK2	PAK group	2.79E-11	LFD	NetworKIN

**Table 2:** List of metabolic enzymes with downregulated phosphosites.

UNIPROT ID	SITE	GENE NAME	PROTEIN DESCRIPTION	LOG FOLD CHANGE	FDR
D3YUK4	S21	NDUFB10	NADH dehydrogenase 1 beta subcomplex subunit 10	−2.84644	0.06
D3YWI1	S46	ALDOA	Fructose-bisphosphate aldolase	−1.1455	0.02
D3YYI5	T185	GM7293	Glyceraldehyde-3-phosphate dehydrogenase	−1.28977	0.01
D3Z1Z2	S79	ACSBG1	Long-chain-fatty-acid–CoA ligase ACSBG1 (Fragment)	−1.3143	0.00
D3Z7K3	S81	ABHD5	1-acylglycerol-3-phosphate O-acyltransferase ABHD5	−2.70938	0.08
E9Q4M2	S964	LIPE	Hormone-sensitive lipase	−2.22608	0.00
P19096	T976	FASN	Fatty acid synthase	−2.49298	0.01
P21550	Y44	ENO3	Beta-enolase	−2.656	0.07
P35486	S293	PDHA1	Pyruvate dehydrogenase E1 component subunit alpha	−1.55234	0.00
P50136	S334	BCKDHA	2-oxoisovalerate dehydrogenase subunit alpha	−1.84521	0.00
P50136	S344	BCKDHA	2-oxoisovalerate dehydrogenase subunit alpha	−3.54903	0.00
P51881	T84	SLC25A5	ADP/ATP translocase 2	−1.60139	0.02
Q05920	S981	PC	Pyruvate carboxylase, mitochondrial	−1.8856	0.02
Q80W21	S67	GSTM7	Glutathione S-transferase Mu 7	−3.09025	0.00
Q91V92	S1090	ACLY	ATP-citrate synthase	−3.97462	0.00
Q91V92	S653	ACLY	ATP-citrate synthase	−2.31841	0.00
Q91V92	S455	ACLY	ATP-citrate synthase	−1.43548	0.00
Q91V92	S455	ACLY	ATP-citrate synthase	−1.5408	0.00
Q91V92	S455	ACLY	ATP-citrate synthase	−2.31553	0.00
Q93092	S237	TALDO1	Transaldolase	−1.05461	0.01
Q9D0F9	T115;S117	PGM1	Phosphoglucomutase-1	−1.6288	0.00
Q9QXG4	S263;S267	ACSS2	Acetyl-coenzyme A synthetase, cytoplasmic	−4.66568	0.00
Q9QXG4	S263	ACSS2	Acetyl-coenzyme A synthetase, cytoplasmic	−2.35304	0.00
Q9QXG4	S265	ACSS2	Acetyl-coenzyme A synthetase, cytoplasmic	−2.67187	0.09
Q9QXG4	S267	ACSS2	Acetyl-coenzyme A synthetase, cytoplasmic	−2.89549	0.00
Q9QXG4	S30	ACSS2	Acetyl-coenzyme A synthetase, cytoplasmic	−1.01133	0.00
Q9QXG4	S30	ACSS2	Acetyl-coenzyme A synthetase, cytoplasmic	−1.72835	0.00
Q9QXG4	S30	ACSS2	Acetyl-coenzyme A synthetase, cytoplasmic	−1.97938	0.00
Q9R0Q7	S148;S151	PTGES3	Prostaglandin E synthase 3	−2.12365	0.00
Q9WVL0	S181	GSTZ1	Maleylacetoacetate isomerase	−2.85077	0.04
Q9Z1E4	S412	GYS1	Glycogen [starch] synthase, muscle	−1.95052	0.03
Q9Z1E4	S645	GYS1	Glycogen [starch] synthase, muscle	−2.03131	0.00
V9GWS1	S47;S49	ACACA	Acetyl-CoA carboxylase 1	−3.42496	0.00
V9GWS1	S47	ACACA	Acetyl-CoA carboxylase 1	−3.90682	0.00
V9GWS1	S23;S29	ACACA	Acetyl-CoA carboxylase 1	−2.81326	0.00
V9GWS1	S23	ACACA	Acetyl-CoA carboxylase 1	−1.64467	0.00
V9GWS1	S29	ACACA	Acetyl-CoA carboxylase 1	−2.6459	0.00
V9GWS1	S79	ACACA	Acetyl-CoA carboxylase 1	−2.1929	0.00
