# Erratum: Phosphoprotein network analysis of white adipose tissues unveils deregulated pathways in response to high-fat diet

**DOI:** 10.1038/srep27230

**Published:** 2016-06-10

**Authors:** Asfa Alli Shaik, Beiying Qiu, Sheena Wee, Hyungwon Choi, Jayantha Gunaratne, Vinay Tergaonkar

Scientific Reports 6: Article number: 2584410.1038/srep25844; published online: 05162016; updated: 06102016.

The original version of this Article contained an error in the spelling of the author Asfa Alli Shaik which was incorrectly given as Alli Shaik Asfa. This has now been corrected in the PDF and HTML versions of the Article.

