# Supplementary material for: Phosphoprotein network analysis of white adipose tissues unveils deregulated pathways in response to high-fat diet
Source: Sci Rep. 2016 May 16;6:25844. doi: 10.1038/srep25844 (PMC4867603; doi:10.1038/srep25844)
Supplement: Supplementary Information [file srep25844-s1.pdf]

Supplementary Information

Phosphoprotein network analysis of white adipose tissues unveils deregulated pathways in response to high-fat diet

Asfa Alli Shaik[†1], Beiying Qiu[†1], Sheena Wee[1], Hyungwon Choi[1,2], Jayantha Gunaratne[*1,3] and Vinay Tergaonkar*[1,4,5]

[1]Institute of Molecular and Cell Biology, Agency for Science, Technology and Research, 61 Biopolis Drive, Singapore 138673, Singapore

[2]Saw Swee Hock School of Public Health, National University of Singapore and National University Health System, 12 Science Drive 2, Singapore 117549, Singapore

[3]Department of Anatomy, Yong Loo Lin School of Medicine, National University of Singapore, 10 Medical Dr, Singapore 117597, Singapore

[4]Department of Biochemistry, Yong Loo Lin School of Medicine, National University of Singapore, Singapore 117597, Singapore

[5]Centre for Cancer Biology, University of South Australia and SA Pathology, Adelaide, Australia.

[†] These authors equally contributed to this work.

*Corresponding author: e-mail: jayanthag@imcb.a-star.edu.sg & vinayt@imcb.a-star.edu.sg

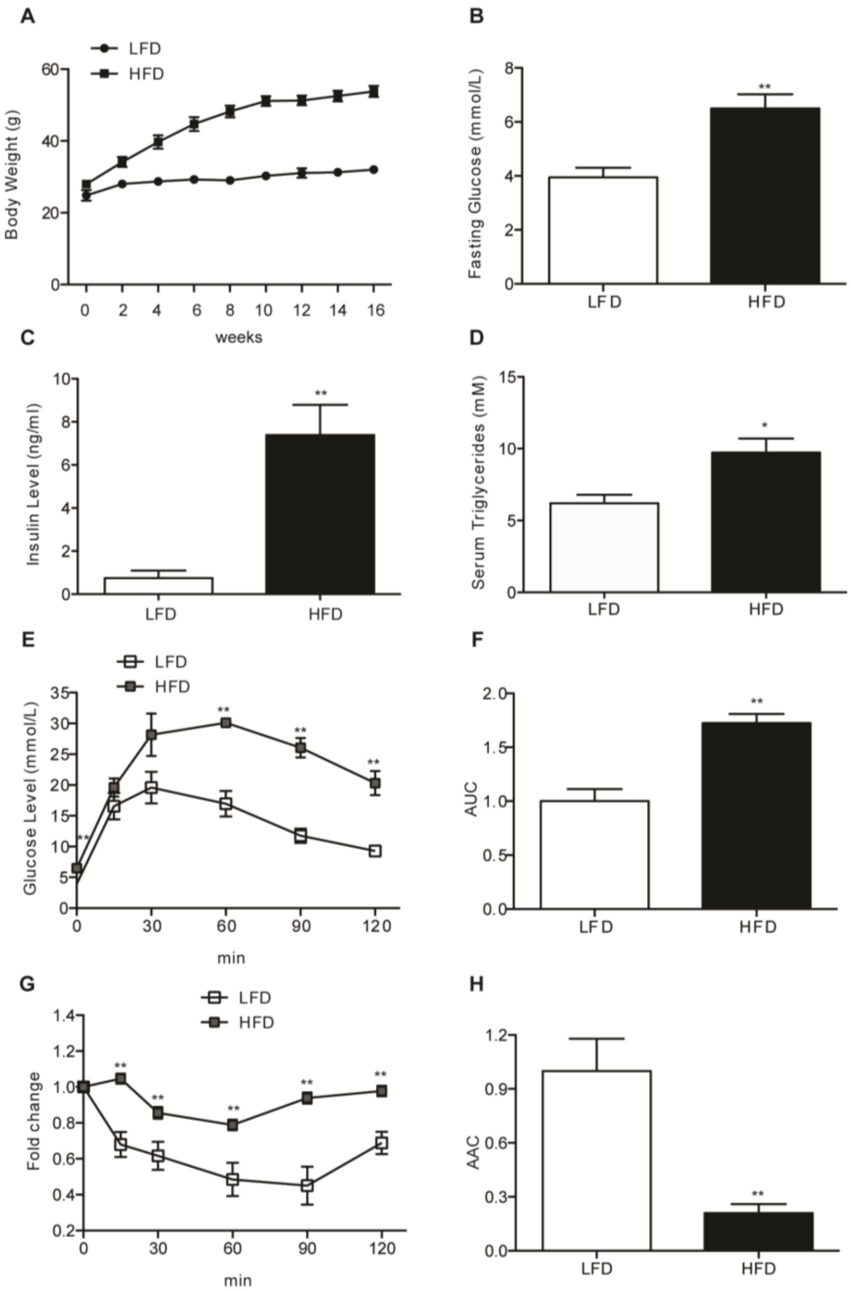

Supplementary Figure 1| Mice under HFD developed obesity and insulin resistance. (A) Body weights of mice fed on HFD and LFD for 4 months (n=10). **(B)** Serum parameters including fasting glucose, **(C)** fasting insulin level, and **(D)** serum triglyceride of mice fed on HFD and LFD for 4 months (n=6). **(E-F)** Glucose tolerance test (GTT) and **(G-H)** insulin tolerance test (ITT) tests were performed on mice fed on HFD and LFD for 4 months (n = 6). AUC (area under curve) and AAC (area above curve) were derived from the GTT and ITT data, respectively. Bars indicate mean ± SE. * $p < 0.05$, ** $p < 0.01$.

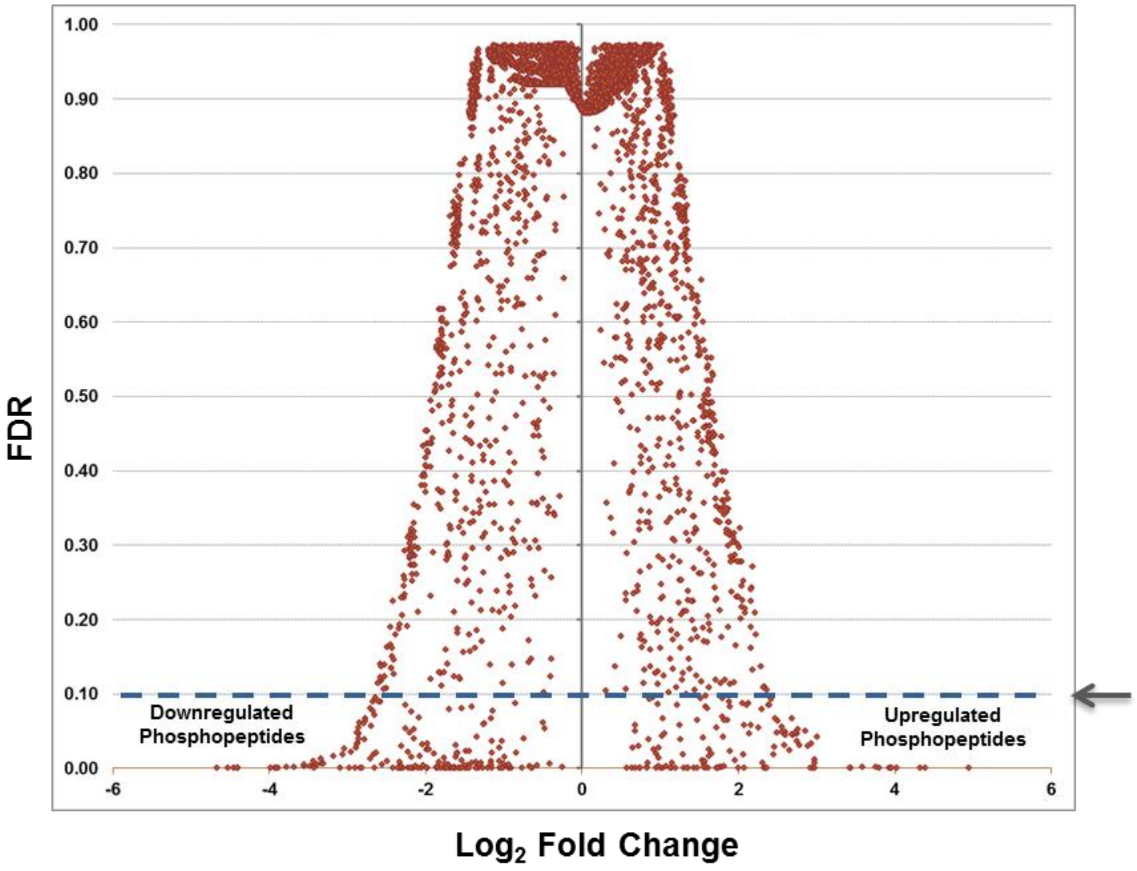

Supplementary Figure 2| Differential phosphopeptide abundances between HFD- and LFD-fed mice. Spectral count-based FDR from phosphopeptides confidently identified at 1% false localization rate using LuciPHOR algorithm were plotted as a function of the spectral count-based fold change between HFD- and LFD-fed mice. The differentially expressed phosphopeptides at 10% FDR is shown below the dotted cut-off line.

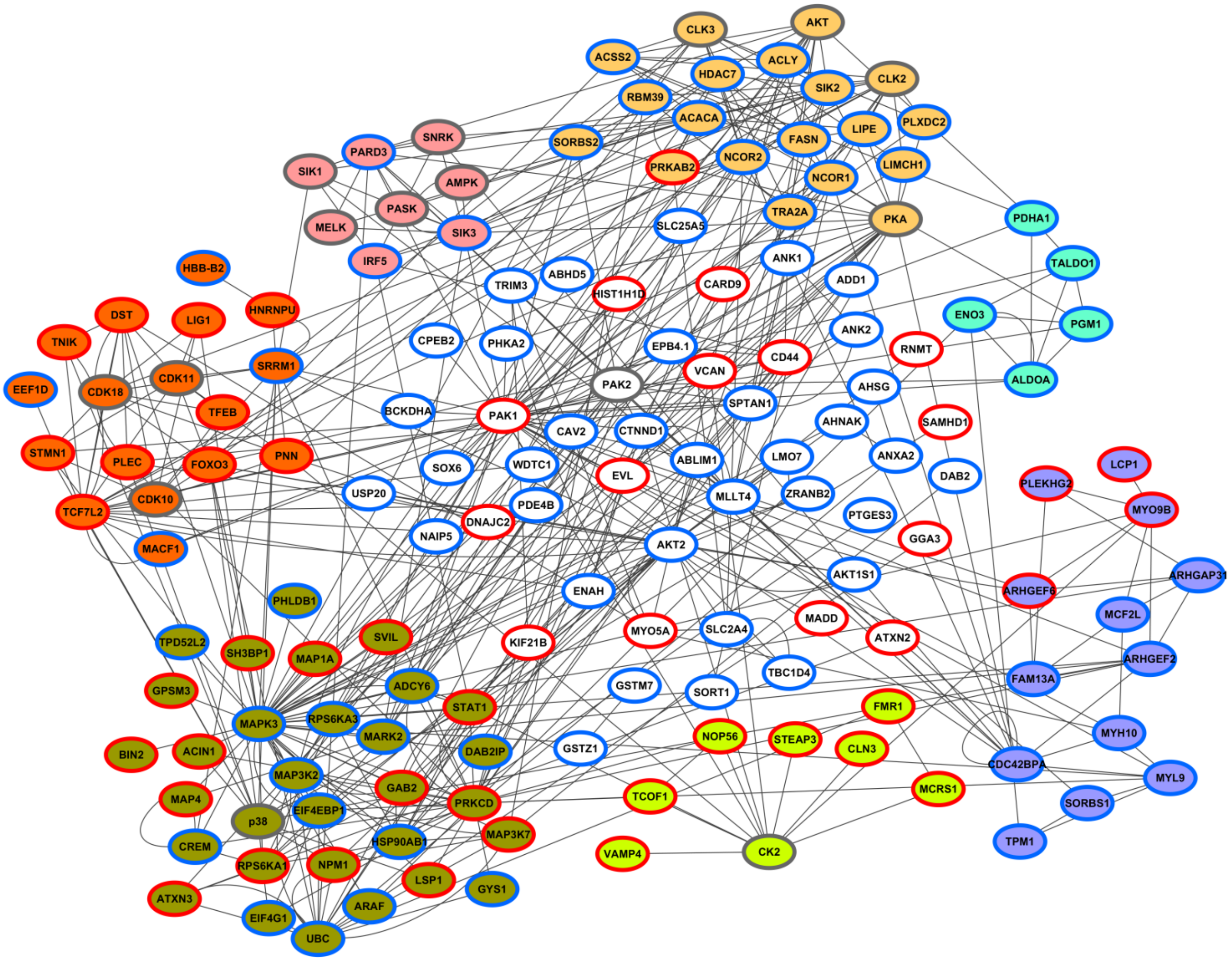

Supplementary Figure 3| Protein-protein interaction network among altered phosphoproteins. Integrated functional and physical protein-protein interaction as identified by STRING, Reactome FI and MetaCore are represented. Interactions associated with predicted kinase-substrate relationships are also shown. Nodes with red and blue borders correspond to upregulated and downregulated phosphoproteins, respectively. Gray borders correspond to predicted kinases. Proteins are grouped according to their clusters enriched using ClusterONE analysis. The total network represents 151 nodes and 476 edges.

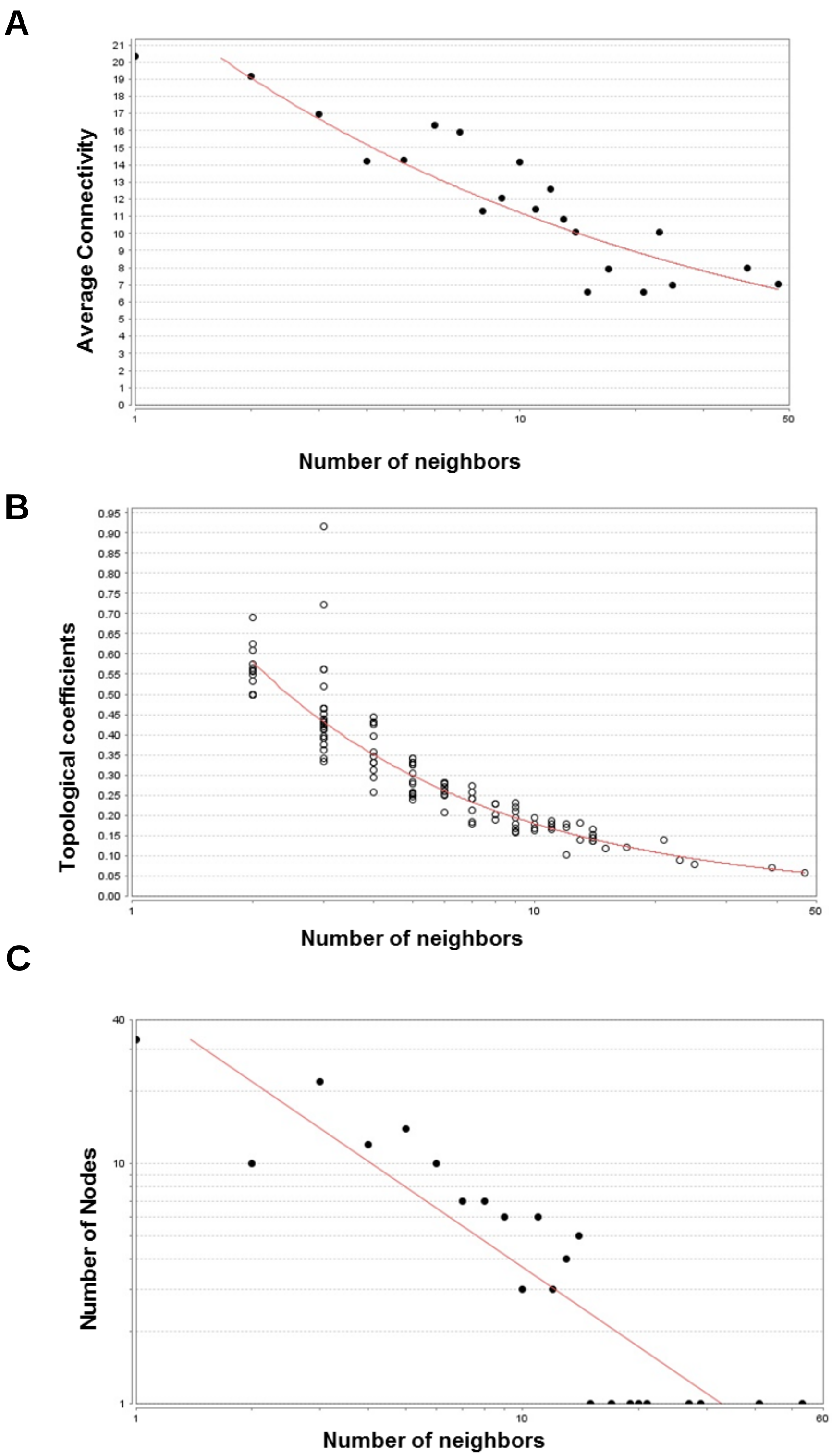

Supplementary Figure 4| Topological properties of network. The integrated protein-protein interaction network follows a scale-free topology as revealed by the power law distribution of node degree as a function of **(A)** average connectivity (R^2= 0.777), **(B)** topological coefficients (R^2= 0.912), and **(C)** node distribution (R^2= 0.821).

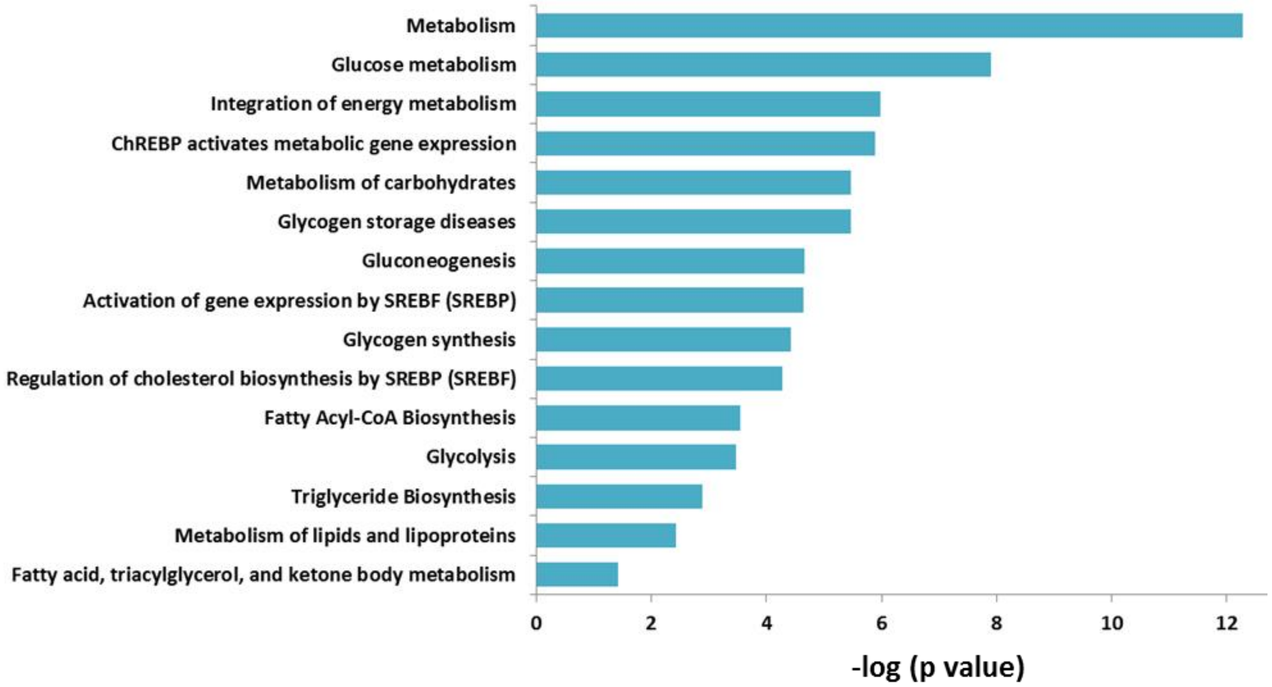

Supplementary Figure 5| Functional groups enriched in dephosphorylated enzymes. Metabolic enzymes with phosphosites showing at least 2-fold downregulation at 1% FDR were subjected to pathway enrichment using Reactome pathway dataset. Over-represented groups with at least 3 proteins are shown.

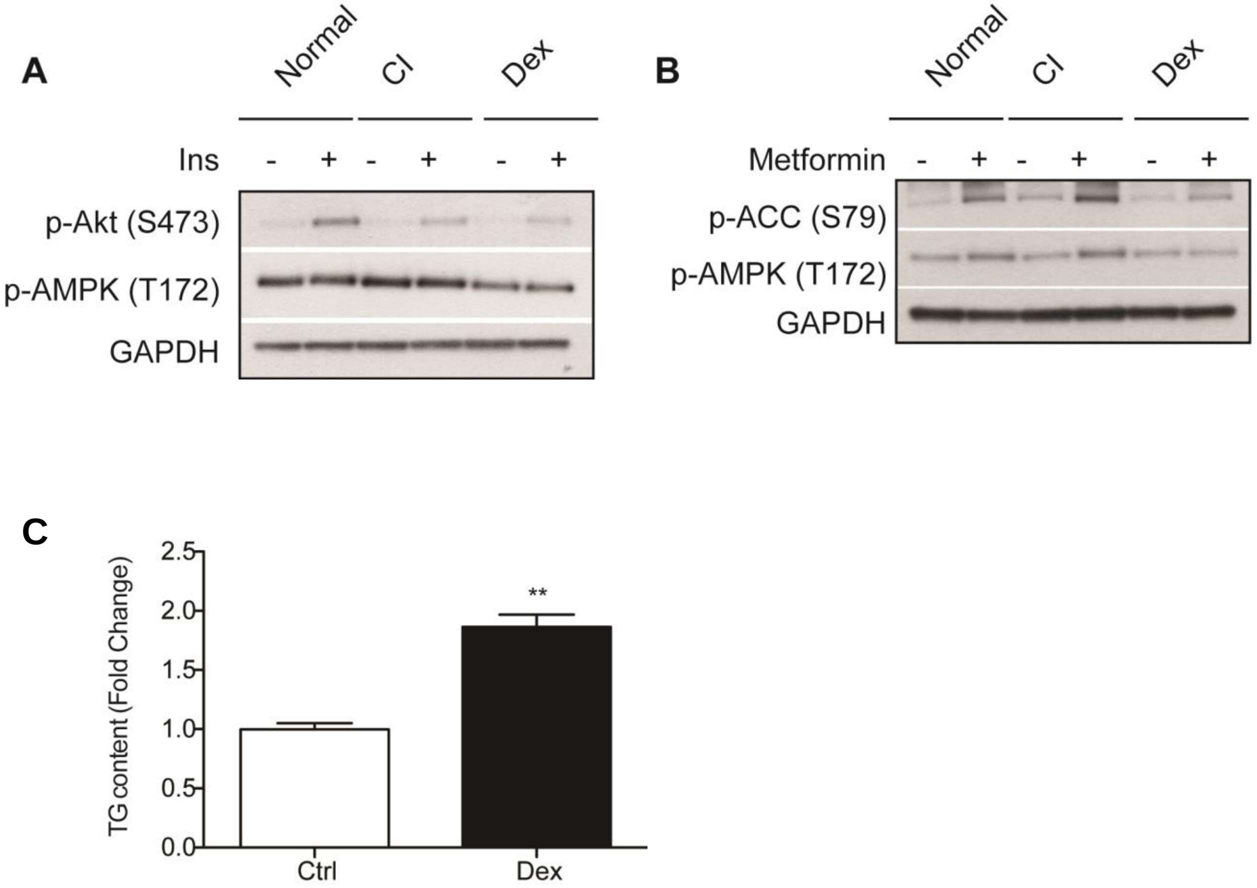

Supplementary Figure 6| Characterization of *in vitro* insulin resistance model. Western blot analysis of chronic insulin (10 nM) or Dex (1 μM) treated 3T3-L1 cells differentiated into adipocytes to assess insulin resistance. After 16 hours treatment, cells were starved in serum-free DMEM for 2 hours followed by **(A)** 100 nM insulin stimulation for 20 minutes or **(B)** by 1 mM metformin for 3 hours. Insulin signaling was checked using p-AKT (S473) and AMPK signaling was assessed using p-AMPK (T172) and p-ACC (S79). GAPDH served as loading control. GAPDH served as loading control (n= 2 biological replicates). **(C)** Fold increase in triglyceride content in 3T3-L1 adipocytes with Dex treatment. Bars indicate mean ± SE. Data are representative of n= 3 biological replicates. ** $p < 0.01$.
